# Wide field-of-view fluorescence imaging for organ-level lineage tracing of rare intestinal stem cell populations

**DOI:** 10.1117/1.JBO.28.9.096004

**Published:** 2023-09-13

**Authors:** Noelle Daigle, Suzann Duan, Heyu Song, Natzem Lima, Ricky Sontz, Juanita L. Merchant, Travis W. Sawyer

**Affiliations:** a University of Arizona, Wyant College of Optical Sciences, Tucson, Arizona, United States; b University of Arizona, College of Medicine, Tucson, Arizona, United States

**Keywords:** fluorescence imaging, lineage tracing, ZBP-89, Zfp148

## Abstract

**Significance:**

Lineage tracing using fluorescent reporters is a common tool for monitoring the expression of genes and transcription factors in stem cell populations and their progeny. The zinc-binding protein 89 (ZBP-89/Zfp148 mouse gene) is a transcription factor that plays a role in gastrointestinal (GI) stem cell maintenance and cellular differentiation and has been linked to the progression of colon cancer. While lineage tracing is a useful tool, it is commonly performed with high-magnification microscopy on a small field of view within tissue sections, thereby limiting the ability to resolve reporter expression at the organ level. Furthermore, this technique requires extensive tissue processing, which is time consuming and requires euthanizing the animal. Further knowledge could be elucidated by measuring the expression of fluorescent reporters across entire organs with minimal tissue processing.

**Aim:**

We present the application of wide-field fluorescence imaging for whole-organ lineage tracing of an inducible Zfp148-tdTomato-expressing transgenic mouse line to assess the expression of ZBP-89/Zfp148 in the GI tract.

**Approach:**

We measured tdTomato fluorescence in *ex vivo* organs at time points between 24 h and 6 months post-induction. Fluctuations in tdTomato expression were validated by fluorescence microscopy of tissue sections.

**Results:**

Quantification of the wide field-of-view images showed a statistically significant increase in fluorescent signal across the GI tract between transgenic mice and littermate controls. The results also showed a gradient of decreasing reporter expression from proximal to distal intestine, suggesting a higher abundance of ZBP-89 expressing stem cells, or higher expression of ZBP-89 within the stem cells, in the proximal intestine.

**Conclusions:**

We demonstrate that wide-field fluorescence imaging is a valuable tool for monitoring whole-organ expression of fluorescent reporters. This technique could potentially be applied *in vivo* for longitudinal assessment of a single animal, further enhancing our ability to resolve rare stem cell lineages spatially and temporally.

## 1 Introduction

Lineage tracing is a widely used technique in biology that enables active monitoring of the migration, proliferation, and differentiation of labeled cell populations *in vivo*.^1^ During normal embryonic development and in certain disease states, stem cell populations arising from the three germ layers migrate and differentiate into specialized progeny cells that support essential physiological functions. Hence, tracking the fate of specific cell populations informs both normal and aberrant organ development and can elucidate the cellular origins of diseased tissues, including cancer.^2^ Modern lineage tracing techniques rely on cell type-restricted expression of fluorescent proteins to label these cell populations and their progeny. Various labeling methods exist and include DNA or viral transfection,^3^ gene-targeting (e.g., Cre-lox and CRISPR/Cas9),^4^^,^^5^ and more advanced techniques, such as DNA barcoding,^6^^,^^7^ and various -omics technologies.^8^^,^^9^ Generally, these labeling methods integrate a traceable reporter, for example, fluorescent proteins or unique barcoded sequences, to the cell populations of interest that can then be detected with high sensitivity and specificity. The method of detection can vary based on the lineage tracing modality used, but most commonly relies on microscopy to detect the expression of fluorescent reporter proteins in frozen tissue sections.^10^^,^^11^ In this case, tissues are removed and briefly fixed in order to cross-link proteins and then are frozen in a cryopreserving compound prior to sectioning and imaging. Under appropriate illumination conditions, fluorescent reporter proteins can be excited and visualized at high resolution, while maintaining some level of physiological context and structure.

Lineage tracing using fluorescence microscopy has contributed an enormous amount of information on cellular origin in different organs and diseases, many of which have been summarized in recent review articles.^1^ One specific application of lineage tracing that is particularly valuable is the ability to spatiotemporally resolve rare stem cell populations through the expression of putative cell-specific transcripts.^12^^–^^14^ For example, the zinc-binding protein 89 kDa ZBP-89 (mouse geneZfp148/human geneZNF148) is a Kruppel-type zinc finger transcription factor that plays a role in gastrointestinal (GI) stem cell maintenance and cellular differentiation.^15^^,^^16^ Our previous studies using an inducible (CreERT2) Zfp148-tdTomato transgenic mouse line showed that ZBP-89 marks intestinal and colonic stem cells and ZBP-89 expression contributes to the development of colon adenomas in mice.^17^

While lineage tracing has clearly proven to be a useful tool, the use of high resolution microscopy is associated with inherent limitations.^18^ First, the ability to generalize observations to whole organs is narrow as a direct result of the small field of view and requirement for tissue processing and sectioning. Thus, tissue and organ-level expression of fluorescent reporter proteins cannot be evaluated directly. Further knowledge of biological mechanisms could be elucidated by measuring the expression of labeled cell populations across entire organs to determine cell behavior and protein expression on a macroscopic scale.^19^ One possible solution is to leverage a technique similar to whole slide imaging to obtain both a microscopic and macroscopic view of fluorescent expression. In this case, the specimen would be imaged using an epifluorescent microscope on an XY displacement stage to acquire tiles over the entire spatial area with some degree of overlap. These tiles are later digitally stitched to create one large composite image.^20^ While this method is capable of creating detailed images of large samples, some limitations include both the long duration of such an acquisition, the potential for extremely large image sizes, and possible limitations of the stage range of motion (e.g., maximum travel distance). In addition, with whole organ imaging, the tissues surface can have significant departure from flat, which would introduce challenges with focusing using a microscopic lens that has a shallow depth of field and is optimized for a thin section of tissue. Methods such as these may be more appropriate for imaging small sets of samples rather than the entire murine GI tract, which has a significantly larger surface area.

Secondly, an added challenge is that fluorescence microscopy also requires extensive tissue processing that involves fixation, embedding, freezing, and sectioning, which is time-consuming and can introduce artifacts to the imaging data.^21^ For example, tissue fixation can quench the intensity of fluorescent protein reporters while simultaneously increasing tissue autofluorescence and introducing greater signal-to-noise ratios with the potential to significantly confound results.^22^ Therefore, an approach to evaluate fluorescent reporter proteins with minimal tissue processing would be advantageous to avoid these challenges. Finally, quantification of fluorescence microscopy images is challenging and is often limited to cell counting and qualitative observations.^23^^,^^24^ Enabling more advanced quantitative methods could enhance the application of lineage tracing and introduce greater rigor to these studies.

To address the challenges associated with lineage tracing of fluorescent reporter proteins, we aimed to develop and test an imaging technology for the rapid and quantitative assessment of an established fluorescent lineage tracing model. Generally, fluorescence imaging can be implemented in a variety of optical system configurations ranging from microscopic to macroscopic.^25^ Wide field-of-view fluorescence imaging instruments are commonly used with targeted fluorescent dyes for surgical guidance and endoscopic surveillance.^26^^,^^27^ Adapting these techniques for whole-organ lineage tracing could address the limitations associated with fluorescence microscopy and enable further knowledge to be gained through lineage tracing. Ultimately, such technology could augment current fluorescence microscopy protocols by enabling rapid, large area assessment of tissue specimens, with the potential for additional *in vivo* application.

Here, we demonstrate the use of wide-field fluorescence imaging for whole-organ detection of rare tdTomato-expressing intestinal stem cells in our tamoxifen-induced Zfp148-CreERT2-tdTomato mouse model. We introduced linear mixed effects (LMEs) models as a method for quantitative assessment of fluorescent signals when assessing multiple, potentially cross-correlated variables. In a first study, we measured the expression of tdTomato by ZBP-89+ cells throughout the GI tract in *ex vivo* tissues over a time span of 3 months following reporter induction, finding that gradients in expression exist both spatially (with higher expression occurring in the proximal intestine) and temporally (tdTomato expression increases over time), consistent with ZBP-89 labeling a stem cell population arising from the epithelial crypts. We then conducted a second study with a higher resolution imaging system to test improved resolving capability and to evaluate fluorescent protein reporter expression up to 6 months post tamoxifen induction. From this, we confirmed the reproducibility of our results and show that it is possible to resolve fine tissue architecture using this approach and visualize individual villi expressing ZBP-89+ progeny. We validated our findings using fluorescence microscopy imaging of frozen sections that were prepared from the same tissues and imaged macroscopically. Ultimately, these results demonstrate that fluorescence imaging can be used on a wide-field scale to assess the expression of fluorescent reporter proteins over a large area that requires minimal tissue processing, and provides robust and reproducible quantitative measurement. This potentially could be applied *in vivo* to enable true longitudinal lineage tracing by evaluating the same specimen over time without sacrifice or tissue removal.

## 2 Methods

We conducted two separate longitudinal studies; study 1 was characterized by a high sample size for statistical power, while study 2 was characterized by higher system resolution to better assess the spatial distribution of fluorescence using wide field-of-view fluorescence imaging. In the first study, we measured tdTomato reporter fluorescence at 24 h, 1 week, 3 weeks, 6 weeks, and 3 months following tamoxifen-mediated induction of the Cre recombinase enzyme (CreERT2). From the results of this initial study, we then sought to develop a higher resolution imaging instrument to determine how well the signal can be localized and resolved using a wide-field approach. In the second study, we further aimed to evaluate whether the tdTomato signal remained up to 6 months post-induction, to support the thesis that ZBP-89 labels a rare intestinal stem cell population that gives rise to specialized epithelial cells throughout the luminal GI tract. It should be noted that no mice were imaged at the 6-month time point in study 1 as we aimed to reproduce our previously reported results, which demonstrated fluorescence up to 3 months and it was not known whether the signal persisted to 6 months.^17^ Study 1 was powered for statistical significance to measure fluorescence at time points ranging up to 3 months and included wild-type controls for each time point. In the second study, we collected images of transgenic mice at all earlier time points, in addition to the 6-month post-induction time point. However, for the second study, Cre-negative littermate control mice were only taken at 6 months for statistical power. No wild-type mice were included for other time points in study 2 as the primary goals of imaging at the earlier time points in this experiment was reproduce observations of study 1 and to qualitatively assess spatiotemporal gradients using high resolution imaging within the tissue, rather than confirm the presence of fluorescent reporters with statistical significance (as was already done in study 1). Table 1 shows the number of mice imaged for each study.

**Table 1:** Sample size for the two imaging studies conducted to evaluate whole-organ lineage tracing. Table entries are specified as (number of wild-type mice/number of transgenic mice).

	1 day	1 week	3 weeks	6 weeks	3 months	6 months
Study 1: statistical power	(4/4)	(5/4)	(3/3)	(4/4)	(2/4)	(0/0)
Study 2: high resolution	(0/2)	(0/2)	(0/2)	(0/2)	(0/2)	(5/5)

### 2.1 Mouse Model and Necropsy

The Zfp148-CreERT2: Loxp-STOP-Loxp tdTomato mouse line was previously reported.^17^^,^^28^ In brief, a transgenic mouse line was generated using a plasmid containing a bacterial artificial chromosome expressing 250 kb of the Zfp148 promoter inserted upstream of the CreERT2 cassette. The Zfp148CreERT2 line was then bred to the Loxp-STOP-Loxp ROSA-tdTomato mouse line purchased from Jackson Labs to generate the Zfp148CreERT2; loxpSTOPloxp-tdTomato hybrid line. A “loxp-STOP-loxp” site was introduced downstream of the Zfp148 promoter sequence and upstream of the tdTomato promoter to drive the Cre-mediated expression of tdTomato protein in ZBP-89 expressing cells. The Cre recombinase was activated by administering 2 mg of tamoxifen (T5648, Millipore Sigma, Burlington, Massachusetts, United States) in 0.1 mL sterile corn oil (C8267, Millipore Sigma) by gastric gavage once. The age of each mouse at the time of tamoxifen injection was variable between roughly 3 and 7 months of age. However, the gut epithelium is expected to turn over approximately every 3 to 5 days,^29^ providing rationale for basing analysis on time since induction rather than initial starting age of each mouse. Littermate controls and tdTomato-expressing Zfp148-CreERT2 mice were euthanized by CO2 inhalation, and organs were explanted, flushed, and the luminal organs were dissected and arranged such that the luminal side was facing the illumination source. All tissues were maintained on ice in phosphate buffered saline (PBS). Images were collected to measure the expression of tdTomato-expressing ZBP-89+ cells in the GI tract, stomach, and liver at the time points, as shown in Table 1. The animal study was reviewed and approved by IACUC Protocol 18–440 PHS Animal Welfare Assurance Number: D16-00159 (A-3248–01) the University of Arizona.

### 2.2 Imaging Systems

**Fig. 1:**
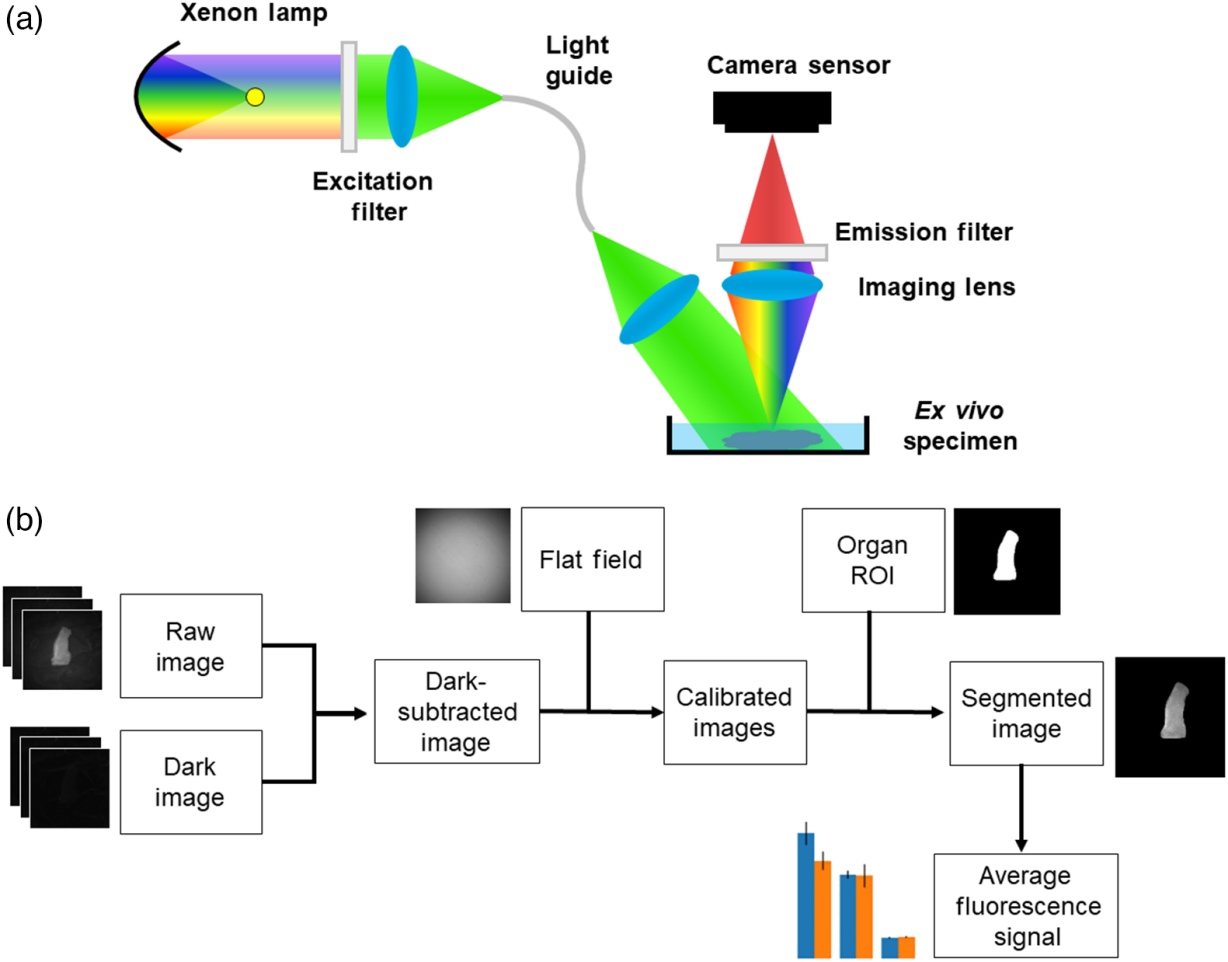
(a) System diagram of the wide-field fluorescence imaging systems used in the two studies and (b) diagram of the data analysis procedures.

Figure 1(a) shows a schematic diagram of the wide-field fluorescence imaging systems used in the reported studies. Both studies used a similar system architecture for detection and illumination. The peak excitation of the tdTomato fluorophore is 554 nm, and peak emission is 581 nm. In general, the excitation and emission spectra for tdTomato are broad, allowing for a degree of flexibility on both the illumination and detection end.^30^ For both studies, illumination was provided by a 300W xenon arc lamp source (LS-OF30, Sutter instruments, Novato, California, United States). Illumination light was filtered with a 561  nm±20  nm bandpass filter (12-152, Edmund Optics, Barrington, New Jersey, United States) and delivered using a fiber bundle (LLG, Sutter Instruments) with a collimation lens. For study 1, images were collected with a 35 mm fixed focal length lens (59-872, Edmund Optics) focusing onto a 0.5-inch CCD Monochrome Camera (EO-1312M, Edmund Optics). The resolution for this system was ∼20  microns, and the integration time was 60 ms. A 594 nm longpass filter (BLP01-594R-25, Semrock) was used to collect emission light. The imaging system used in study 2 was reported and characterized in detail previously.^25^ The illumination system is identical to study 1, but in this case, the imaging camera is a high performance back-illumination CMOS sensor (QHY600M, QHY, Beijing, China), coupled with a photographic lens (NIKKOR 55 mm f/2.8, Nikon, Tokyo, Japan) to produce high performance imaging. The resolution for this system was ∼7  microns, and the integration time was 20 ms. The vignetted appearance of images from study 2 is due to the limited numerical aperture of the illumination light guide being unable to fill the entire field of view of the sensor. To ensure high quality data acquisition, we used a brightfield image as a reference during data acquisition to orient all tissues such that they were within the illuminated region before beginning fluorescence imaging acquisitions. In future iterations of this system, this issue could be corrected by integrating additional illumination optics to expand the numerical aperture.

In both cases, the detection system was mounted on a rigid arm above the sample plane. Images were saved in 16 bit .tif format. Prior to imaging, a white diffuse reflectance target (58-609, Edmund Optics) was imaged to correct for spatial non-uniformity using unfiltered illumination. Additionally, a power measurement was collected for the filtered illumination light at the sample plane using a power meter (S120C, Thorlabs, Newton, New Jersey, United States).

### 2.3 Data Analysis

Data were analyzed identically for both studies, diagrammatically, as shown in Fig. 1(b). First, using reflectance images as reference, regions of interest were manually drawn for each organ using ImageJ to create a binary mask corresponding to the organ area of interest. These binary masks were saved as separate image files.^31^ Masks were created for stomach, liver, and the GI tract, including duodenum, jejunum, ileum, and colon.

All acquired images were dark subtracted, normalized by the flat field image to correct for illumination non-uniformity, and scaled by the light source power. The binary masks were then applied separately for each organ and the mean value of the region of interest was calculated to assess fluorescence intensity. Statistical comparisons were conducted between wild-type and transgenic mice for each time point using a two-tailed t-test.

To assess potentially overlapping and mixed effects of different biological variables,––namely the sex of the subject and whether the reporter was homozygously expressed,—we used LMEs modeling to evaluate which variables influence expression of the fluorescence reporter.^32^^,^^33^ We first aimed to estimate the effect of time on the reporter expression to confirm the results found using the t-test. To do so, we can model the measured fluorescence intensity using standard LME notation in the following equation:

F∼T+C(S)+C(H), (1)

where F is the dependent variable, which is the measured fluorescence intensity; T is a quantitative variable referring to the time since injection in weeks; C(S) is a categorical variation referring to the sex of the subject; and C(H) is a categorical variable referring to whether the subject homozygously expresses the reporter. Data were grouped according to the organ and a random intercept was applied to each group. We next applied LME to evaluate the expression as a function of organ, along with effects due to sex and homozygosity. The equation to do so is shown in the following equation:

F∼C(O)+C(S)+C(H), (2)

where C(O) is a categorical variable referring to the organ, which has possible values of duodenum, jejunum, ileum, colon, and liver, whereas F, C(S), and C(H) are as before. Here data were grouped according to the time point and a random intercept was applied to each of these groups.

For the second study, we confirmed our results for organ-level expression using LME for the expression, as shown in Eq. (3). Note that the subject distribution did not contain sufficient numbers of homozygous and heterozygous subjects to include this effect. Here, all samples were grouped according to time point with a random intercept for each group

F∼C(O)+C(S). (3)

All LMEs models created and optimized using Python 3 (Python Software Foundation, Beaverton, Oregon, United States) with the statsmodel package. All models were optimized using restricted maximum likelihood and were found to converge. In building these models, wild-type mice and stomach tissues were removed from the analysis, as we are investigating the effects within the positive experimental group.

### 2.4 Tissue Fixation and Fluorescence Microscopy

Immediately following wide-field fluorescence imaging using our systems, all tissues were fixed in 4% paraformaldehyde for 30 min prior to dehydrating overnight at 4° in a 30% sucrose-PBS solution. Tissues were embedded in optical cutting temperature (OCT) compound (Sakura Finetek USA, Torrance, California, United States) and frozen at −80°C. OCT-embedded tissues were sectioned at −20°C to a 10-micron thickness using a TN50 semi-automatic cryostat (Tanner Scientific, Sarasota, Florida, United States). Frozen sections were adhered onto glass slides, then mounted with a #1.5 glass coverslip using Prolong Gold Anti-fade mounting medium containing 4’,6-diamidino-2-phenylindole (Life Technologies, Rockville, Maryland, United States). Slides were imaged using the same acquisition (exposure time) settings for the tdTomato channel at 200× magnification using an Olympus BX53F epifluorescence microscope (Center Valley, Pennsylvania, United States). This microscopic fluorescence imaging serves as a gold standard reference for the wide-field fluorescence imaging completed in studies 1 and 2.

## 3 Results

### 3.1 3 Month High Sample Size Study

**Fig. 2:**
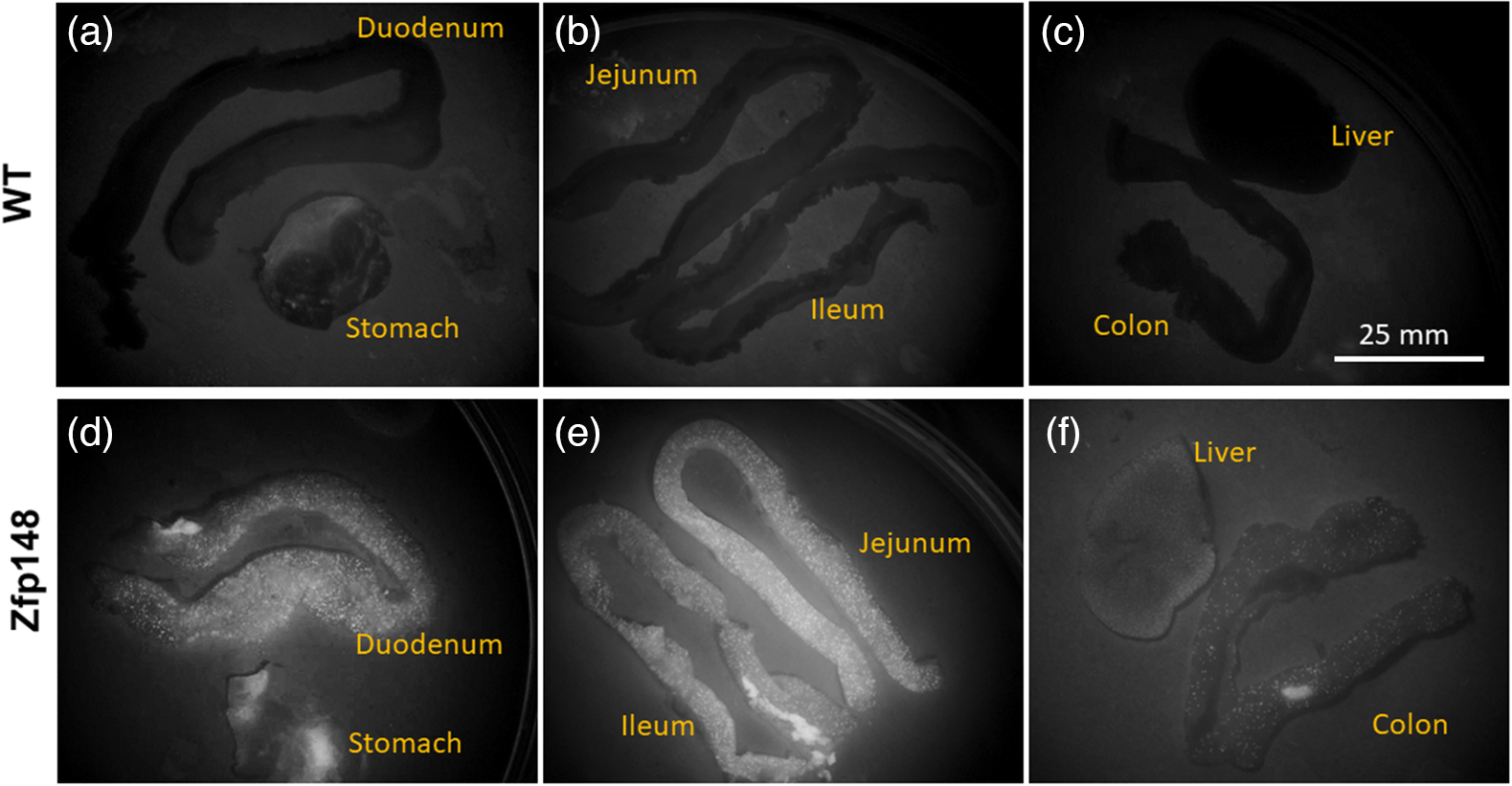
Example images of whole-organ fluorescence imaging taken during study 1 for various murine organs taken at 3 months for wild type [WT; (a)–(c)] and transgenic [Zfp148; (d)–(f)]. Qualitatively, there is a strong signal in transgenic mice compared to WT mice throughout the gastrointestinal tract. Autofluorescence is observed in the stomach for both transgenic and WT littermate control mice. Brightness of all images shown was digitally increased by 40% and contrast was increased by 20% for better visualization.

Our results confirm that ZBP-89 is expressed ubiquitously in the GI tract in our transgenic line. Representative raw fluorescence images from 3-month mice in study 1 are shown in Fig. 2, which illustrate significant expression of the fluorescent reporter is visible using wide-field imaging. The signal appears to manifest as bright spots with a diffuse background. As expected, wild-type mice have almost no detectable signal.

**Fig. 3:**
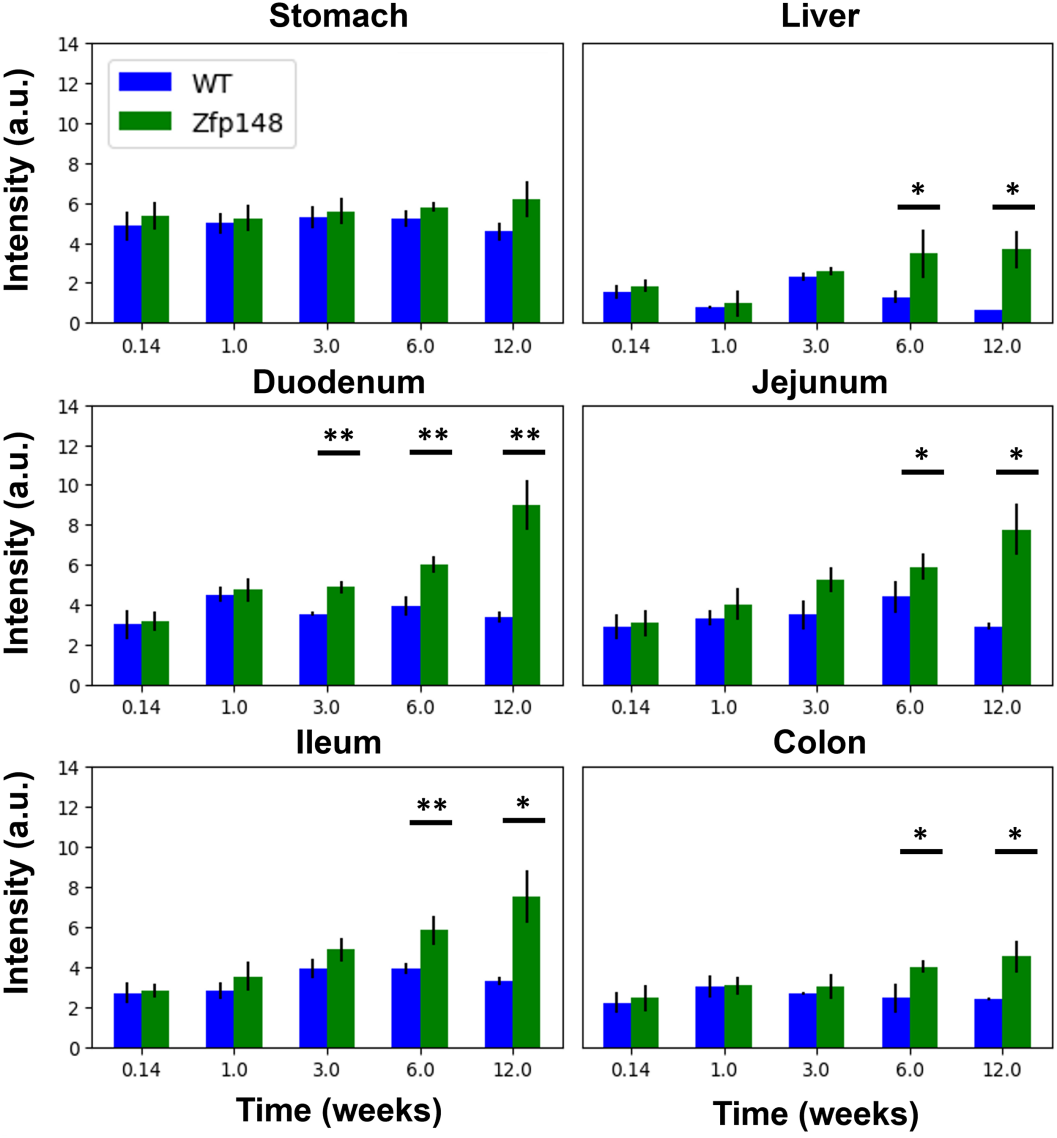
Quantification of wide-field fluorescent images in study 1, showing an increase over time in fluorescent signal in transgenic mice compared to wild-type littermate controls for all GI tissues except stomach. Error bars denote standard deviation. Sample sizes are listed in Table 1. Significance is denoted as * (p<0.05) and ** (p<0.01) using a two-tailed t-test.

**Fig. 4:**
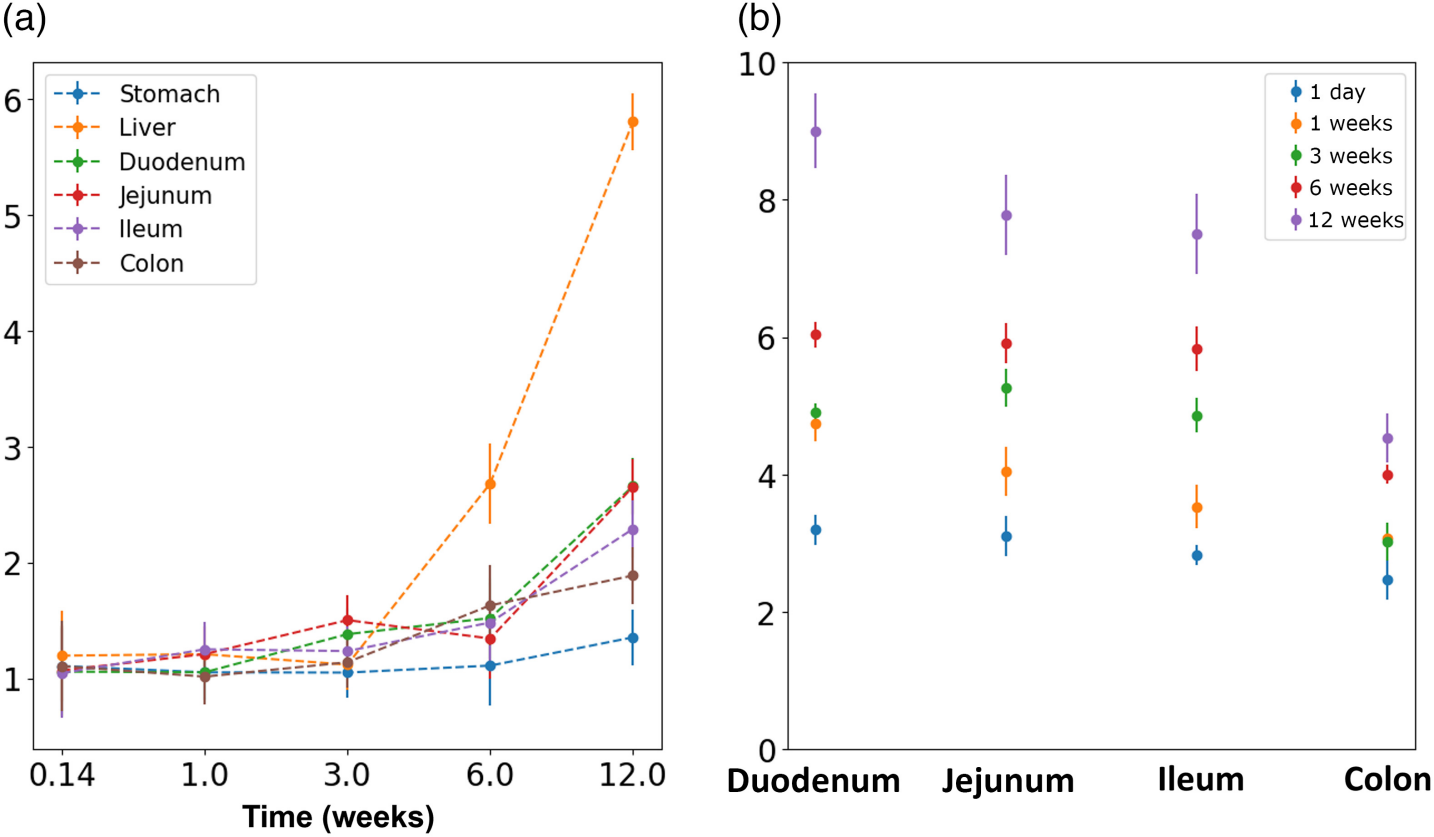
(a) Fluorescent signal relative to wild type as a function of time shows consistent increase from 1 day to 12 weeks. (b) At all time points, a gradient of higher to lower tdTomato expression across the proximal to distal GI tract was observed. Sample sizes are listed in Table 1. Error bars denote standard deviation. Duo – duodenum and Jej – jejunum.

Quantification of the images, as shown in Fig. 3, illustrates a statistically significant increase of fluorescent signal across the GI tract between transgenic and wild-type mice. There were no significant differences observed in the stomach tissues, and differences in signal with other tissues become more significant at longer time points, which indicates the label becomes increasingly expressed as time goes on. These results also suggest a gradient of decreasing expression from the proximal to distal intestine, which suggests a higher abundance of ZBP-89+ stem cells and differentiated progeny cells in the proximal intestine. We also note there is no significant signal at the 24 h time point as anticipated as mRNA transcription, translation, and protein folding may take longer than 24 h and up to 72 h.^34^ Thus, there is likely no fluorescent protein being produced by 24 h. In transgenic mice, Fig. 4(a) shows the collected signal relative to wild type as a function of time, and Fig. 4(b) shows the acquired fluorescent signal as a function of tissue in the GI tract, further supporting the observations of spatial and temporal gradients.

To quantitatively assess the potential dependence of expression on these parameters, LME models were used. Table 2 summarizes the results from the LME model including effects from time (in weeks), sex, and homozygosity of tdTomato sequences on both alleles. The results show a highly significant (p<0.001) dependence on time and tdTomato homozygosity. The positive coefficient for time post-induction indicates that the expression will increase with time, as was qualitatively observed in Fig. 4(a), and the positive coefficient for homozygosity indicates that homozygous subjects will have an increase in expression compared to heterozygous mice with a single tdTomato allele. There was no significant dependence on sex as a biological variable for ZBP-89-tdTomato expression. The intercept parameter would imply that there is a nonzero offset at the first time point, which would be influenced by tissue autofluorescence levels and any observed reporter expression.

**Table 2:** LMEs model results for model fitted to Eq. (1). This models the effect of time, sex, and homozygosity on the fluorescence reporter expression. Results are shown as LME coefficient, standard error, Z-score, and statistical significance denoted as *** for p<0.001. Sex coefficient refers to male relative to female. Homozygosity refers to mice with genotyping positive for floxed tdTomato sequences on both alleles relative to heterozygous mice in which one allele is the wild type for tdTomato expression (negative). Upper and lower confidence intervals (C.I.) are shown for 95% confidence.

	Coefficient	Standard error	Z scores	Significance	C.I. 2.5%	C.I. 97.5%
Intercept	3.248	0.750	4.331	***	1.778	4.718
C (sex) (M)	−0.204	0.255	−0.798	n.s	−0.703	0.296
C (homozygosity) (Y)	1.460	0.283	5.162	***	0.906	2.015
Time (week)	0.160	0.012	13.288	***	0.137	0.184
Group variance	2.582	1.654	—	—	—	—

Similarly, Table 3 summarized results for the LME model built to assess effects of organ location and cross-effects of sex and homozygosity. Compared to the first LME model, we observe a similar result for the overall intercept and the homozygosity parameter (p<0.001), which supports the previous finding. We also see that there is no significant difference due to sex for this model, again confirming the previous result. From this LME model, we see that we obtain highly significant effects due to organ location. Coefficients are shown relative to signal obtained in the colon, and we see that the duodenum has the highest increase, followed by jejunum and ileum, (all p<0.001) consistent with the qualitative observations made in Figs. 3 and 4(b). The coefficient for the liver is negative, which indicates a lower overall signal, and confirms the apparent relationship observed in Fig. 3.

**Table 3:** LMEs model results for model fitted to Eq. (2). This models the effect of organ, sex, and homozygosity on the fluorescence reporter expression. Results are shown as LME coefficient, standard error, Z-score, and statistical significance denoted as * for p<0.05 and *** for p<0.001. Sex coefficient refers to male relative to female. Homozygosity refers to homozygous relative to heterozygous. All organ coefficients are shown relative to colon. Upper and lower confidence intervals (C.I.) are shown for 95% confidence.

	Coefficient	Standard error	Z scores	Significance	C.I. 2.5%	C.I. 97.5%
Intercept	2.838	0.735	3.86	***	1.398	4.278
C (sex) (M/F)	0.336	0.268	1.25	n.s.	−0.190	0.862
C (homozygosity) (Y/N)	1.708	0.304	5.62	***	1.112	2.304
C (organ) (duodenum)	3.065	0.363	9.45	***	2.429	3.701
C (organ) (jejunum)	2.522	0.324	7.77	***	1.886	3.158
C (organ) (ileum)	2.044	0.313	6.30	***	1.409	2.68
C (organ) (liver)	−0.774	0.301	−2.39	*	−1.410	−0.139
Group variance	2.723	1.570	—	—	—	—

### 3.2 6 Month High-Resolution Study

**Fig. 5:**
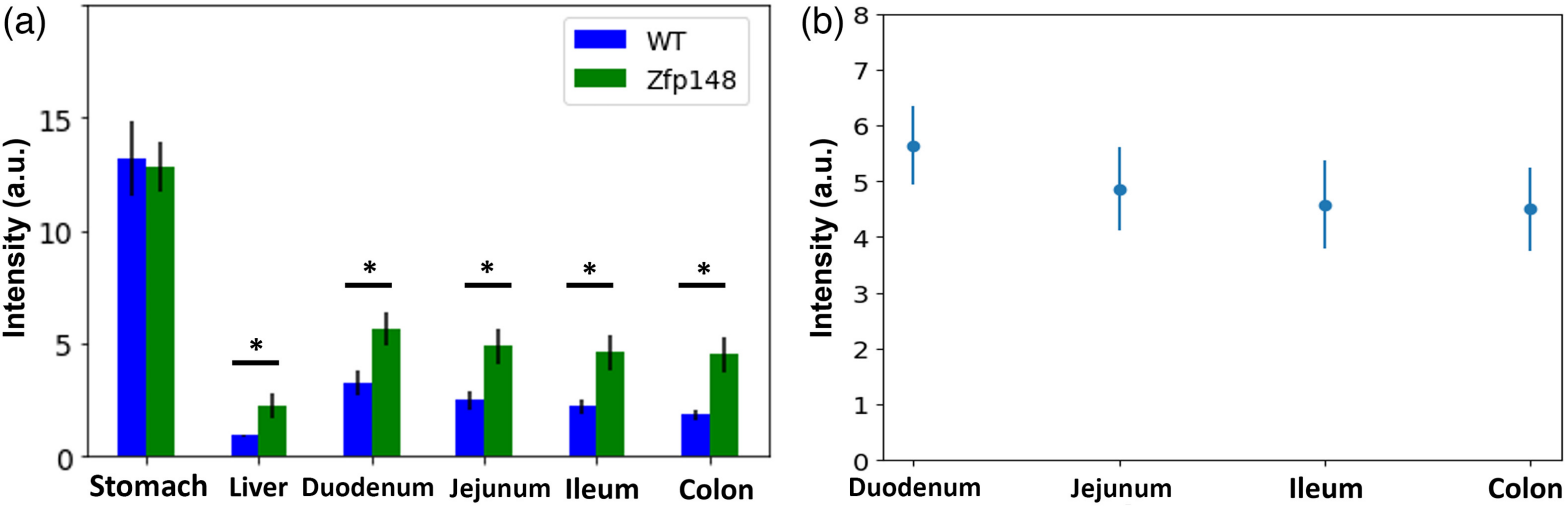
(a) Fluorescent signal relative to wild type at the 6 month time point, showing a similar trend of expression across organ locations as in study 1. n=5 for each Zfp and WT. (b) Fluorescence intensity in transgenic samples as a function of organ location, showing decrease from the proximal to distal GI tract. Error bars denote standard deviation. Duo – duodenum and Jej – jejunum.

Figure 5(a) shows quantitative results from the high-resolution imaging study for fluorescent signal collected from transgenic and wild-type mice at 6 months post-induction. Note that as the earlier time points for study 2 were limited to two transgenic samples each and no wild-type control, these time points were not powered for statistical significance and instead were primarily used for qualitative analysis. The overall relationship of expression is consistent with the first study, strongly supporting the reproducibility of the results. As before, we see significant increases in tdTomato fluorescence for transgenic mice in every organ except the stomach. Figure 5(b) shows the signal as a function of organ location at 6 months, once again following the decreasing expression gradient from proximal to distal intestine that was originally observed.

Table 4 summarizes results obtained from the LME model used to quantitatively evaluate signal as a function of organ location, as well as assess cross-effects of subject sex. Note that compared to study 1 (Tables 2 and 3), the coefficient scale will vary due to differences in detection system, such as different dynamic ranges and sensitivity. As with study 1 (shown in Table 4), we see no significant difference due to sex, and the results are also consistent in that the proximal intestine (e.g., duodenum) illustrates the highest signal increase compared to the distal intestine (e.g., colon), with a gradient in between. Note that compared to study 1, the coefficient scale will vary due to differences in detection system due to different dynamic ranges and sensitivity.

**Table 4:** LMEs model results for model fitted to Eq. (3). This models the effect of organ and sex on the fluorescence reporter expression for mice at 6 months post-induction in study 2. Results are shown as LME coefficient, standard error, Z-score, and statistical significance denoted as * for p<0.05 and *** for p<0.001. Sex coefficient refers to male relative to female. All organ coefficients are shown relative to colon. Upper and lower confidence intervals (C.I.) are shown for 95% confidence.

	Coefficient	Standard error	Z scores	Significance	C.I. 2.5%	C.I. 97.5%
Intercept	6.654	0.531	12.537	***	5.614	7.694
C (sex) (M/F)	−0.548	0.310	−1.768	n.s.	−1.155	0.060
C (organ) (duodenum)	1.413	0.530	2.665	***	0.374	2.452
C (organ) (Jejunum)	1.362	0.586	2.323	*	0.213	2.511
C (organ) (Ileum)	1.087	0.555	1.958	*	−0.001	2.174
C (organ) (liver)	−2.708	0.591	−4.583	***	−3.867	−1.550
Group variance	0.852	0.066	—	—	—	—

Taking both studies together, the use of LME models proves to be a powerful tool for the analysis and interpretation of this data. To make impactful conclusions from complex biological models, it is important to assess whether, and how, different variables influence the expression of markers. This approach could be applied generally for other models and labeled markers to evaluate the combined effects of multiple variables.

**Fig. 6:**
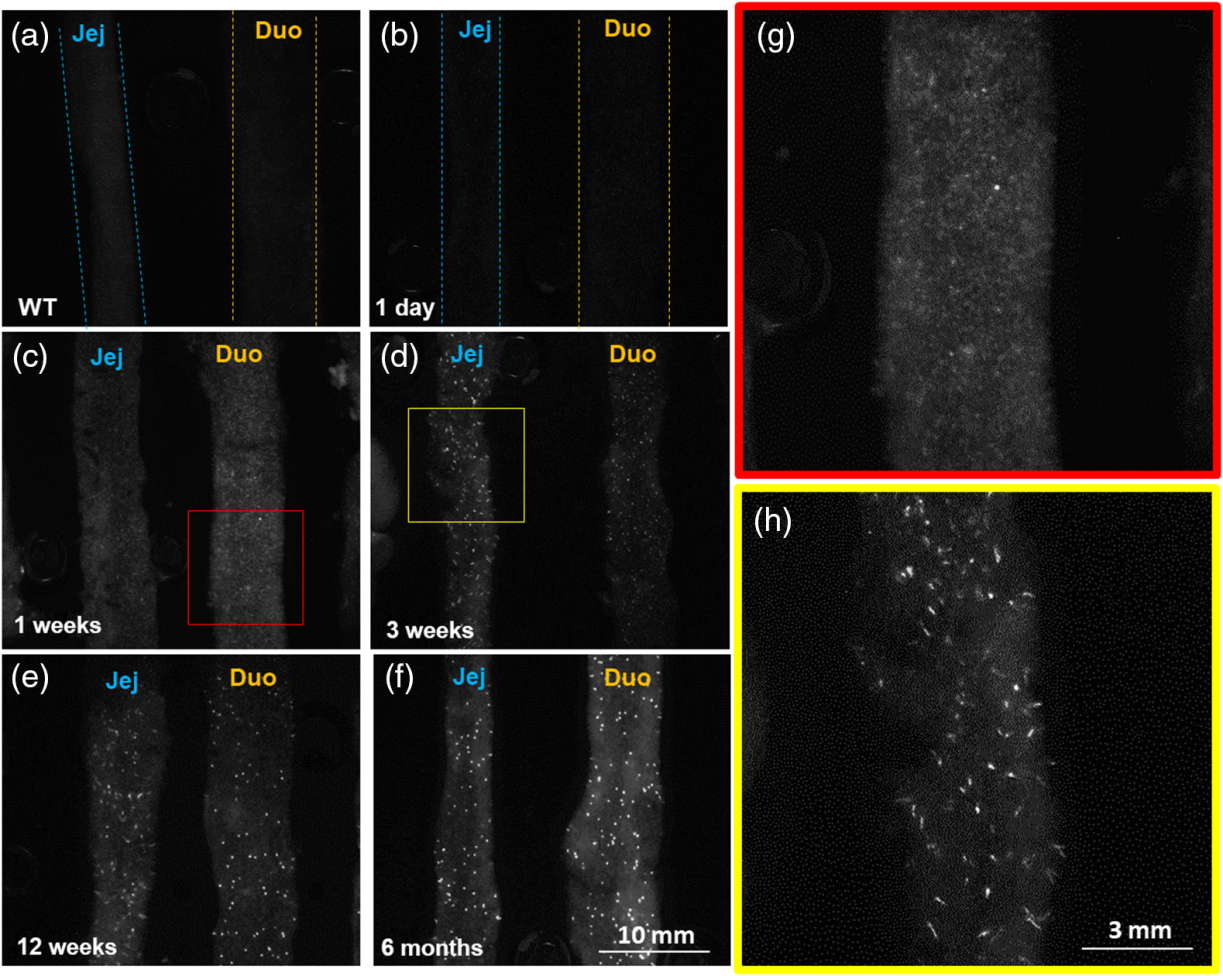
Wide-field fluorescence images collected in study 2 for the duodenum and jejunum. (a) Wild-type mice up to 6 months and (b) transgenic mice 1 day post-induction show no noticeable signal. (c) Transgenic mice at 1 week post-induction show a diffuse distribution of signal, and the signal begins to concentrate into localized regions at (d) 3 weeks, (e) 12 weeks, and (f) 6 months. (g) Higher resolution inspection of the diffuse signal at 1 week [red box in panel (c)] shows texture patterns to the signal, whereas at (h) 3 weeks, the signal is localized to small thin regions that are thought to be the villus. Jej – jejunum and duo – duodenum. Panel (a) is the only wild type image and (b)–(h) transgenic mice at different time points.

Figure 6 shows wide-field images collected for the duodenum and jejunum in the high-resolution imaging study to illustrate the sensitivity of tdTomato detection. Figure 6(a) shows that wild-type animals have negligible signal and that transgenic mice at one day post injection [Fig. 6(b)] are similar in expression levels. At 1 week, fluorescence begins to appear as diffusely distributed across the organ [Fig. 6(c)]. As time progresses, we observed punctate expression of fluorescent signal that grows in intensity over time [3 weeks, 12 weeks, and 6 months, as shown in Figs. 6(d)–6(f), respectively]. Inspecting the resolving capabilities of the system in the early signal at 1 week [Fig. 6(g)] appears to have some minor texture to the pattern but few concentrated strong areas of signal. This may suggest that the markers are expressed broadly as cells differentiate across the organ. As time progresses, for example, 3 weeks [Fig. 6(h)], higher resolution inspection of the punctate pattern reveals that these bright sources of signal have long thin hair-like projections, consistent with the villus tissue structure of the luminal GI tract. This finding suggests that the cells expressing ZBP-89 differentiate and/or migrate up the length of the villus with time, but these cells may originate in stem-cell like populations in the crypt zones deeper in the tissues, giving rise to the early diffuse signal.

**Fig. 7:**
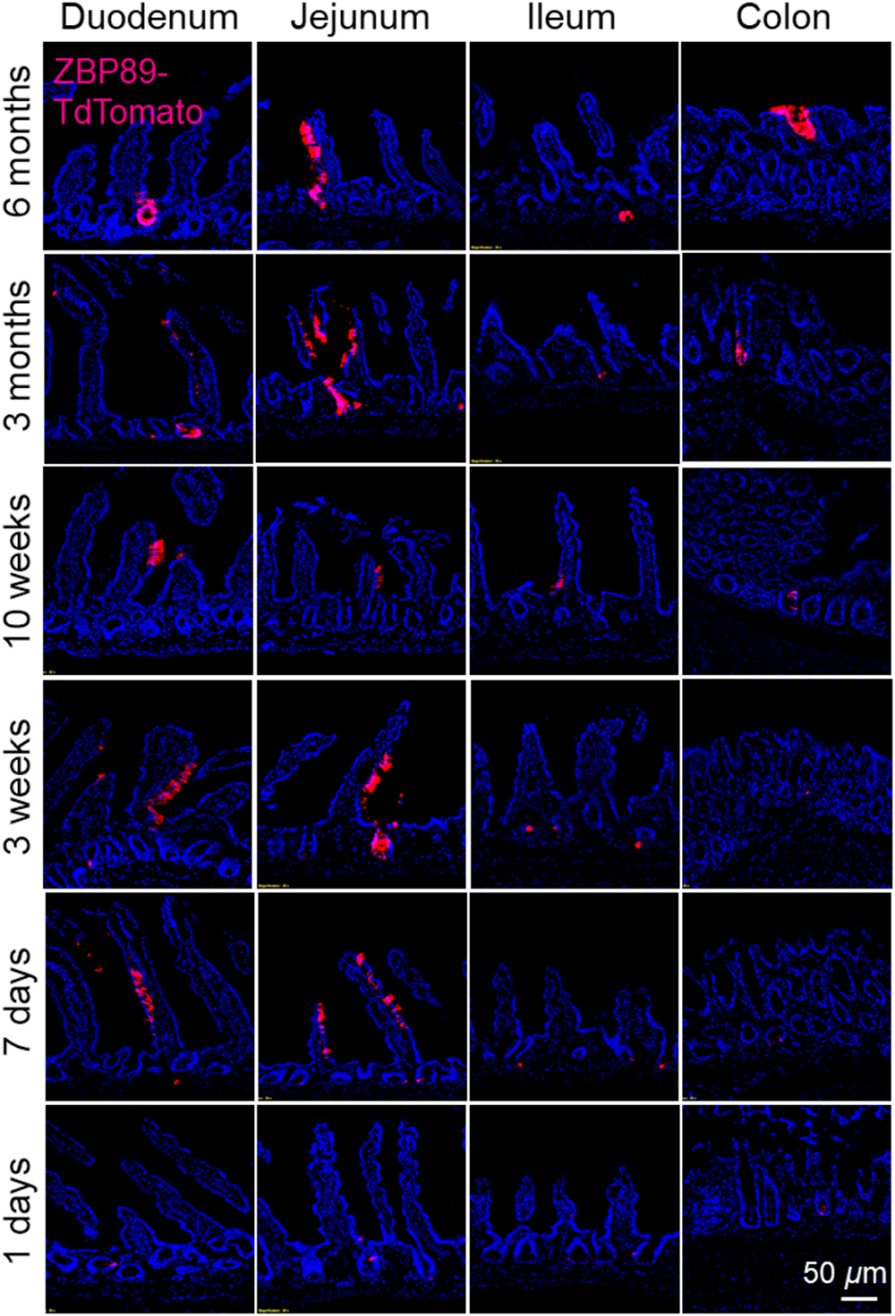
Fluorescence microscopy images of frozen tissue sections from transgenic mice previously imaged using wide-field fluorescence imaging. These images were taken with an Olympus BX53F epifluorescence microscope with a 200× objective. TdTomato expression is consistent between wide-field and microscopic images with a gradient of higher to lower expression from proximal to distal intestine and increased signal intensity at longer time points following Cre recombinase induction with tamoxifen.

To confirm these findings, Fig. 7 shows representative fluorescence microscopy images of cryopreserved tissue sections from our mouse model at each time point. The expression pattern of tdTomato is consistent with our wide-field results. We observe that tdTomato expression occurs more diffusely immediately following Cre recombinase induction, suggesting that ZBP-89 is expressed early on in the crypts of the epithelial mucosa. Over time, the tdTomato signal localizes to long hair-like projections, consistent with the distinct villi microstructure of the luminal mucosa. We also observe increased tdTomato expression in the proximal intestine, suggesting that ZBP-89 is more highly expressed in this organ compared to the stomach and distal GI tract.

**Fig. 8:**
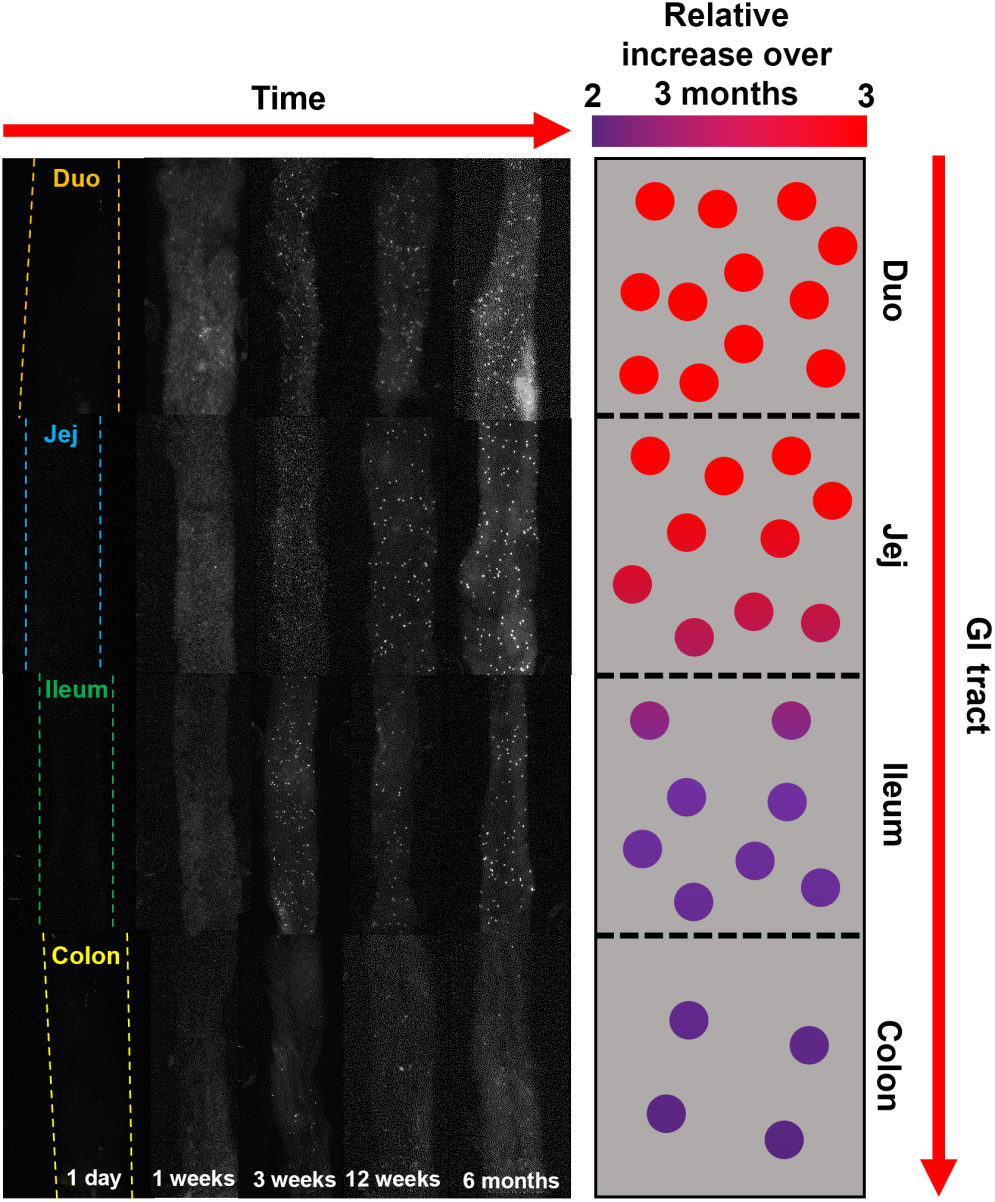
A diagram showing trends in fluorescence in transgenic mice over location and time. The color of the dots indicates increase in signal over time from 1 day to 3 months, and the density of dots indicates fluorescence abundance across all time points relative to the colon. Images were collected during study 2. Duo – duodenum and Jej – jejunum.

Figure 8 is a graphic detailing the aforementioned observed spatiotemporal gradients in transgenic mice. In summary, fluorescence expression increases with time and decreases from the proximal to distal intestine. To the left, representative images from one sample at each time point have been arranged in order of increasing temporal and spatial properties. To the right, an illustration depicts these trends in a simplified form. The color of the dots indicates increase in signal over time from 1 day to 3 months, as indicated by Fig. 4(a), and the density of dots indicates fluorescence abundance across all time points relative to the colon, as calculated in Table 3.

## 4 Discussion

Overall, the findings from these studies demonstrate that wide-field fluorescence imaging could be a useful tool for macroscopic lineage tracing and measuring distributions of fluorescent labels in biological tissues. The advantages of such a technique are multifold: the wide-field approach enables assessment at the organ level, which is not influenced by potential bias introduced by limited field of view and thin tissue sections seen with fluorescence microscopy. The wide-field approach also enables immediate assessment of fluorescent protein expression, whereas tissue processing, such as fixation may result in quenching of the fluorescent reporter and increased tissue autofluorescence. Another advantage is the ease of signal quantification. We introduced LME as a potential method for analyzing multivariate data obtained using this approach, but other quantitative methods could be used as well. For example, we calculate mean fluorescent signal to quantify our image, whereas an alternative could be to implement a type of particle counting to evaluate the number of positive cells or villus in these images. Such quantitative methods need to be developed to address specific biological questions that may exist for a given model.

One limitation of a wide-field approach is the resolution, and therefore specificity, will not be equal to that achieved using microscopy. However, we demonstrate that using state-of-the-art camera sensors, such as that used in the second study, the resolving power is still sufficiently high to resolve small structures, such as murine villus. Furthermore, a major drawback in microscopic lineage tracing is that specimens must be sacrificed at each time point, preventing true lineage tracing, as it is not possible to measure the same specimen or tissue area at each time point so that each animal can serve as its own control. Although we collect *ex vivo* images in these studies, the method could theoretically be translatable *in vivo* using endoscopic imaging techniques, which would enable true lineage tracing. Other advancements in the future could be the integration of multiple fluorescent labels and corresponding filters for multiplexed imaging of different markers in real time. One such way this has been recently implemented in small animal imaging is through the use of the so called “Brainbow” transgenic model, where four fluorophores were originally multiplexed to label different cellular components.^35^ From this model, different transgenic lines have since been developed for multiplexed labeling of different tissues and markers of interest. For example, studies have successfully used this method originally to map neuronal connectivity and, as the technique has developed, to perform lineage tracing for multiple cell populations at once.^36^ Combining this advanced labeling technique with the application of wide-field multispectral fluorescence imaging for measuring spatiotemporal distributions of the fluorophores could provide unique biological information to elucidate the causative mechanisms of disease, among other broad applications in biology.

Through these studies, we obtain several interesting findings using our model. First, we observed spatial and temporal gradients in the expression of ZBP-89 along the intestinal tract and its expression is consistent with restricted expression in a rare stem cell population, as previously reported.^17^ This could either be due to an increase in the number of expressing cells, or an increased concentration of fluorescent proteins within the cells. We also recognize that future studies will be required to fully address whether the population of Zfp148-positive stems cells remains consistent throughout the adult lifespan; our work has suggested but not confirmed that the number of stem cells within the crypts is not increasing with mouse age, as we observed an increase in Zfp148-tdTomato signal in the differentiated epithelium and not in the crypts themselves. We observe that there are no significant differences in expression due to sex; however, hetero- and homozygous expression of the fluorescent reporter sequence significantly determine signal intensity. Furthermore, we showed that our observations with wide-field fluorescence imaging strongly correlated with tdTomato signal frozen sections resolved by fluorescence microscopy, giving confidence that our approach provides insight that could be used to inform future biological investigations on ZBP-89 expressing intestinal stem cells.

## 5 Conclusion

Using the Zfp148 CreERT2-tdTomato mouse model, we demonstrated that wide-field fluorescence imaging is a powerful tool for lineage-tracing rare stem cell populations throughout the GI tract. We show through two separate approaches that the expression of ZBP-89 throughout the intestine increases over 6 months and that its expression forms a gradient with the highest expression observed in the proximal intestine that diminishes towards the distal intestine (ileum) and colon. We applied LME models to evaluate the influence of multiple variables on Zfp148Cre-tdTomato expression, providing statistical confidence in our results. Using a high-resolution imaging system, we then showed that our technique can provide sufficient resolution to observe fine tissue structures in real time, and we validated our findings with fluorescence microscopy of tissue cryosections. These results show promise for this technology as a method for whole-organ lineage tracing and for mapping distribution of fluorescently labeled biological markers. In the future, this technique could potentially be applied *in vivo* for longitudinal assessment of a single animal, further increasing the translation and impact of lineage tracing.

## Code, Data, and Materials Availability

Data and code used for analysis is publicly available in the following FigShare repository: https://doi.org/10.25422/azu.data.24166266
